# Predicting mortality in intensive care unit patients with acute pancreatitis using an interpretable machine learning model

**DOI:** 10.3389/fmed.2025.1592051

**Published:** 2025-07-21

**Authors:** Li Zhuangli, Zhang Xingcheng, Zhang Xiaoli, Lu Zhonghua, Sun Yun

**Affiliations:** ^1^The First Department of Critical Care Medicine, The Second Affiliated Hospital of Anhui Medical University, Hefei, China; ^2^Department of Critical Care Medicine, The 901 Hospital of the Joint Logistic Support Force of the Chinese People’s Liberation Army, Clinic College, Anhui Medical University, Hefei, China; ^3^Department of Critical Care Medicine, Fuyang Second People's Hospital, Fuyang, China

**Keywords:** intensive care unit, acute pancreatitis, mortality, machine learning model, SHapley additive exPlanations

## Abstract

**Background:**

Acute pancreatitis (AP) in the intensive care unit (ICU) is linked to elevated in-hospital mortality rates. Timely identification of high-risk patients remains challenging. This study aimed to develop an interpretable machine learning model for predicting in-hospital mortality in ICU patients with AP and to identify key contributing factors.

**Methods:**

A retrospective analysis was performed on 306 ICU patients diagnosed with AP. After data preprocessing and feature selection via the Least Absolute Shrinkage and Selection Operator (LASSO), seven machine learning models were developed: decision tree, random forest, XGBoost, support vector machine (SVM), multilayer perceptron, k-nearest neighbors (KNN), and logistic regression. Model performance was evaluated using the area under the receiver operating characteristic curve (AUC), Brier score, calibration plots, and decision curve analysis (DCA). The SHapley Additive exPlanations (SHAP) framework was utilized to interpret model predictions and assess feature importance rankings.

**Results:**

Multivariate logistic regression analysis identified the following independent risk factors for in-hospital mortality in ICU patients with AP: acute physiology and chronic health evaluation (APACHE II) score, activated partial thromboplastin time (APTT), albumin (Alb), blood urea nitrogen (BUN), creatinine (Cr), use of vasoactive agents, and ICU length of stay. The AUC values for the seven machine learning models in the training set were DT (0.947), RF (0.900), XGBoost (0.887), SVM (0.901), MLP (0.837), KNN (0.983), and LR (0.876). In the validation set, the corresponding AUC values were DT (0.698), RF (0.850), XGBoost (0.878), SVM (0.892), MLP (0.822), KNN (0.755), and LR (0.858). Although DT and KNN demonstrated high sensitivity and specificity in the training set, their performance was suboptimal in the validation set. SHAP analysis ranked APACHE II score as the most influential predictor of mortality.

**Conclusion:**

An interpretable SVM model incorporating routinely available clinical variables effectively predicts in-hospital mortality in ICU patients with AP. SHAP-enhanced interpretation highlights key predictors and enhances model transparency, supporting clinical decision-making.

## 1 Introduction

Acute pancreatitis (AP) ranks among the most prevalent gastrointestinal conditions that necessitate hospitalization, with incidence rates showing considerable variation across various regions—ranging from about 4.9 to 73.4 per 100,000 patients—and indicating an increasing trend in recent years (1). While most cases of AP are mild with a favorable prognosis, about 20% of patients develop severe acute pancreatitis (SAP), which is characterized by sepsis or multi-organ failure, necessitating admission to the intensive care unit (ICU) (2). In such cases, mortality rates increase markedly, ranging from 17.6 to 52% (3).

Currently, several scoring tools are employed to predict the prognosis of AP, including the Ranson score (4), the Bedside Index for Severity in Acute Pancreatitis (BISAP) (5), and the Computed Tomography Severity Index (CTSI) (6). Each tool has its own advantages and limitations (7). In recent years, advancements in artificial intelligence have facilitated the integration of various machine learning algorithms into the medical field. These algorithms are currently frequently employed in auxiliary diagnosis, prognosis assessment, and survival analysis, making them vital instruments in clinical research (8).

Prominent among these are statistical algorithms such as logistic regression (LR) and machine learning models, including support vector machines (SVM), artificial neural networks (ANN), random forests (RF), and decision trees (DT) (8, 9). Although most current predictive models exhibit high accuracy, they often prioritize model discrimination over interpretability, leading to reluctance among clinicians to trust and utilize these models (10).

Consequently, this study seeks to create machine learning models utilizing different algorithms to predict in-hospital mortality among AP patients in the ICU, determine the most effective model, and improve its interpretability through SHapley Additive exPlanations (SHAP). Ultimately, this study seeked to determine key prognostic factors influencing the outcomes of AP patients in the ICU.

## 2 Materials and methods

### 2.1 Study design

This retrospective single-cohort study was conducted on adult patients diagnosed with acute pancreatitis (AP) who were admitted to the first department of the ICU from September 2013 to September 2023. Data were collected, analyzed, and used to develop predictive models to identify risk factors associated with mortality in ICU-admitted AP patients.

### 2.2 Study population

Inclusion Criteria: (1) Patients aged 18 years and older; (2) Diagnosis of AP confirmed by the following criteria: (a) Abdominal pain indicative of acute pancreatitis, (b) Serum amylase or lipase levels at least three times the normal values; (c) Diagnostic imaging (CT, MRI, or ultrasound) showing characteristic features of acute pancreatitis. Exclusion Criteria: (1) Patients with malignant tumors; (2) Pregnant patients; (3) Variables with over 30% missing data.

### 2.3 Data collection

General patient information was collected, including age, gender, body mass index (BMI), and disease etiology. Various scores (SOFA score, Marshall score, APACHE II score) and laboratory test results were also recorded. All test results were the first obtained within 24 h of ICU admission. The laboratory tests included: Serum amylase (Amy); White blood cell (WBC), percentage of neutrophils (NEUT), C-reactive protein (CRP), procalcitonin (PCT), interleukin-6 (IL-6); Brain natriuretic peptide (BNP); Platelet (PLT), prothrombin time (PT), activated partial thromboplastin time (APTT), fibrinogen (Fib) and D-dimer (D-D); Albumin (Alb), total bilirubin (TB), direct bilirubin (DB), total cholesterol (TC), triglyceride (TG), alanine aminotransferase (Alt), blood urea nitrogen (BUN), creatinine (Cr), blood glucose (Glu), blood calcium (Ca2+) and blood potassium (K+); Interventions (e.g., invasive mechanical ventilation, use of vasoactive agents, CRRT, use of antibiotics or hormones, abdominal puncture drainage, and laparotomy), comorbidities (e.g., cardiovascular disease, hypertension, chronic obstructive pulmonary disease, diabetes mellitus, renal insufficiency), complications (e.g., pancreatic necrosis, sepsis, or septic shock), and outcome measures (e.g., in-hospital mortality, length of hospital stay, length of ICU stay) were also recorded.

### 2.4 Model construction

The dataset was first randomly split into a training set (70%) and a validation set (30%). Variable selection was performed exclusively on the training set using Least Absolute Shrinkage and Selection Operator (LASSO) logistic regression, with in-hospital mortality (1 = death, 0 = survival) as the binary outcome variable. The optimal regularization parameter was determined via 10-fold cross-validation using the lambda.1SE criterion. Candidate predictors identified by the LASSO model (i.e., those with non-zero coefficients) were subsequently entered into a multivariable logistic regression model to further verify their independent association with the outcome. Variables with statistical significance (*p* < 0.05) were retained as final predictors. These selected features were then used as input variables for downstream model construction. To evaluate and compare the predictive performance, we applied seven commonly used machine learning algorithms, including DT, RF, Extreme Gradient Boosting (XGBoost), SVM, Multi-Layer Perceptron (MLP), K-Nearest Neighbors (KNN), and Logistic Regression (LR). For each model, the relative importance of predictors was assessed based on their internal feature weights or contribution metrics.

### 2.5 Model evaluation

Five-fold cross-validation was used for model comparison and hyperparameter selection. Receiver Operating Characteristic (ROC) curves were generated for all datasets, with the corresponding area under the curve (AUC) values calculated to quantify diagnostic performance. A comprehensive evaluation of predictive capability was performed through accuracy, F1 score, and Brier score metrics. Calibration curves were plotted to assess model accuracy in probability estimation, while clinical decision curve analysis (DCA) was implemented to evaluate the net clinical benefit across various threshold probabilities. SHapley Additive exPlanation (SHAP) values were calculated to determine the contribution of each feature to the prediction model, illustrating the impact of individual features. This multimodal assessment framework enabled systematic identification of the optimal predictive model through integrated analysis of discrimination, calibration, and clinical utility parameters.

### 2.6 Statistical method

Statistical analyses were performed using R version 4.2.3 and Python version 3.11.4. Categorical data were presented as counts (n) or percentages (%) and compared between groups using the chi-square test. Normally distributed measurement data were expressed as means ± standard deviation (x ± s) and compared between groups using the independent sample t-test. Non-normally distributed data were presented as medians (M) with first and third quartiles (Q1, Q3) and compared using the Wilcoxon rank sum test. A *p*-value of less than 0.05 was considered statistically significant.

### 2.7 Ethics

This study received approval from the Ethics Committee of the Second Affiliated Hospital of Anhui Medical University (No. YX2023-136).

## 3 Results

### 3.1 Patient characteristics

A total of 306 AP patients were enrolled in the study, including 267 patients in the survival group and 39 in the death group. Compared to the survival group, the death group had a higher proportion of male patients and an older average age. Additionally, more patients in the death group received treatments such as vasoactive agents (VA), mechanical ventilation (MV), glucocorticoids, and continuous renal replacement therapy (CRRT). The death group also had a higher incidence of surgical interventions, abdominal puncture drainage, and complications. APACHE II, Marshall, and SOFA scores were significantly higher in the death group. Serum levels of amylase, procalcitonin (PCT), interleukin-6 (IL-6), B-type natriuretic peptide (BNP), prothrombin time (PT), activated partial thromboplastin time (APTT), D-dimer (D-D), direct bilirubin (DB), blood urea nitrogen (BUN), creatinine (Cr), triglycerides (TG), and total cholesterol (TC) were also elevated in the death group (*p* < 0.05). In contrast, platelet (PLT), fibrinogen (Fib), and albumin (Alb) levels were lower, and ICU stay duration was longer in the death group (all *p* < 0.05). No significant differences were observed in the other indicators between the two groups (Table 1). The dataset was randomly divided into training and validation sets in a 7:3 ratio. The training set comprised 216 cases used for model training, while the remaining 90 cases were utilized for model validation. In-hospital mortality rates were 12.5% in the training set and 13.3% in the validation set. Clinical data comparisons between the survival and death groups, as well as between the training and validation sets, are presented in Table 1.

**Table 1:** Baseline characteristics of patients included.

Variables	Survivors (*n* = 263)	Nonsurvivors (*n* = 43)	All patients (*n* = 306)	*p*	Training set (*n* = 214)	Validation set (*n* = 92)	All patients (*n* = 306)	*p*
Age (years)^a^	49 (38, 66)	57 (46, 71)	50 (38.2, 66.8)	0.030	50 (39, 67)	50 (38, 64.8)	50 (38.2, 66.8)	0.528
Gender^b^				0.011				0.449
Male	141 (52.8)	29 (74.4)	170 (55.6)		123 (56.9)	47 (52.2)	170 (55.6)	
Female	126 (47.2)	10 (25.6)	136 (44.4)		96 (44.4)	41 (45.1)	137 (44.6)	
BMI (Kg/m^2^)^a^	26.4 (24.2, 28)	27.6 (24.6, 29.4)	26.5 (24.3, 28.2)	0.130	26.4 (24.2, 28.1)	26.6 (24.6, 28.2)	26.5 (24.3, 28.2)	0.589
APACHE II score^a^	12 (8.5, 16)	19 (15, 23.5)	13 (9, 18)	< 0.001	12 (9, 18)	13 (9, 17)	13 (9, 18)	0.581
SOFA score^a^	4 (2, 7)	6 (5, 8)	5 (3, 7)	< 0.001	4 (3, 7)	5 (3, 7)	5 (3, 7)	0.471
Modified Marshall score^a^	2 (1, 3.5)	4 (3, 5)	2 (1, 4)	< 0.001	2 (1, 4)	2 (1, 4)	2 (1, 4)	0.777
Comorbidities^b^								
DM				0.169				0.207
No	207 (77.5)	34 (87.2)	241 (78.8)		166 (76.9)	75 (83.3)	241 (78.8)	
Yes	60 (22.5)	5 (12.8)	65 (21.2)		50 (23.1)	15 (16.7)	65 (21.2)	
Hypertension				0.255				0.377
No	213 (79.8)	28 (71.8)	241 (78.8)		173 (80.1)	68 (75.6)	241 (78.8)	
Yes	54 (20.2)	11 (28.2)	65 (21.2)		43 (19.9)	22 (24.4)	65 (21.2)	
Cardiovascular disease				1.000				0.875
No	251 (94)	37 (94.9)	288 (94.1)		203 (94)	85 (94.4)	288 (94.1)	
Yes	16 (6)	2 (5.1)	18 (5.9)		13 (6)	5 (5.6)	18 (5.9)	
COPD				1.000				0.164
No	253 (94.8)	37 (94.9)	290 (94.8)		202 (93.5)	88 (97.8)	290 (94.8)	
Yes	14 (5.2)	2 (5.1)	16 (5.2)		14 (6.5)	2 (2.2)	16 (5.2)	
Chronic renal insufficiency				0.912				0.012
No	221 (82.8)	32 (82.1)	253 (82.7)		171 (79.2)	82 (91.1)	253 (82.7)	
Yes	46 (17.2)	7 (17.9)	53 (17.3)		45 (20.8)	8 (8.9)	53 (17.3)	
Laboratory test^a^								
AMY (U/L)	518 (113, 1, 370)	1, 006 (347.5, 1800.5)	571.5 (143.5, 1, 439)	0.017	604.5 (116.8, 1583.5)	436.5 (177, 1272.8)	571.5 (143.5, 1, 439)	518 (113, 1,370)
WBC (×10^9^/L)	12.8 (8.9, 17)	14.4 (10.8, 20.2)	12.9 (9, 17.3)	0.089	12.8 (8.9, 17)	13.2 (9.5, 17.9)	12.9 (9, 17.3)	0.332
N (%)	85.9 (80.6, 90.3)	86.7 (81.8, 89.8)	86 (80.7, 90.2)	0.455	86.1 (80.7, 90.6)	85.8 (81.3, 89.5)	86 (80.7, 90.2)	0.454
CRP (mg/L)	178.8 (73.1, 265.4)	172.6 (99, 262.2)	176.6 (73.6, 266.4)	0.803	181.9 (96.7, 264.4)	165.9 (63.3, 271.2)	176.6 (73.6, 266.4)	0.527
PCT (ng/ml)	1.5 (0.4, 5.9)	2.5 (1.2, 22)	1.6 (0.5, 6.2)	0.018	1.6 (0.5, 7.2)	1.7 (0.5, 5.4)	1.6 (0.5, 6.2)	0.600
IL-6 (pg/ml)	139.8 (53.7, 461)	234.4 (119.8, 507.4)	161.6 (56, 463.4)	0.02	160.2 (59.2, 451.8)	163.1 (54.3, 631.8)	161.6 (56, 463.4)	0.716
BNP (ng/l)	149 (77.5, 335.5)	297 (121, 752.5)	156 (80.8, 429)	0.017	155.5 (78, 365.8)	162 (86.8, 438.2)	156 (80.8, 429)	0.627
PLT (×10^9^/L)	171 (113.5, 228.5)	129 (88.5, 198)	167.5 (112, 226.8)	0.033	164 (104.5, 225.2)	175 (127, 233.8)	167.5 (112, 226.8)	0.153
PT (S)	12.8 (11.7, 14.5)	13.9 (12.2, 15.7)	12.9 (11.7, 14.7)	0.044	12.9 (11.8, 14.7)	12.8 (11.7, 14.5)	12.9 (11.7, 14.7)	0.926
APTT (S)	30.5 (25.7, 36.5)	35.7 (28.9, 45.4)	31.2 (26.1, 37.6)	0.002	31.2 (26.1, 38)	31 (26.1, 36.7)	31.2 (26.1, 37.6)	0.889
FIB (g/L)	4.9 (3.3, 6.8)	3.3 (2.1, 4.9)	4.7 (3.2, 6.8)	0.002	4.5 (3.2, 6.8)	5 (3.2, 7.1)	4.7 (3.2, 6.8)	0.410
D-D (ug/ml)	3.9 (2, 6.3)	6.6 (3.7, 9.6)	4.1 (2.1, 6.8)	< 0.001	4.3 (2.4, 6.8)	4 (1.7, 6.4)	4.1 (2.1, 6.8)	0.298
Alb (g/L)	28.5 (25.2, 35)	22.6 (18.4, 27)	28.1 (24.5, 34.8)	< 0.001	28.4 (24.8, 35.5)	27.1 (23.6, 32.8)	28.1 (24.5, 34.8)	0.150
TB (umol/L)	21.5 (13, 33.9)	24.7 (19.6, 52.4)	22 (13.3, 36.3)	0.083	22.1 (13.1, 34.8)	22 (13.6, 44.1)	22 (13.3, 36.3)	0.975
DB (umol/L)	7 (3.5, 16.1)	13.6 (5.9, 40.4)	7.8 (3.8, 18.4)	0.006	7.8 (4.2, 17.2)	7.6 (3.2, 19.1)	7.8 (3.8, 18.4)	0.545
TG (mmol/L)	2 (1.1, 6.4)	1.3 (1, 2.2)	1.9 (1.1, 5.5)	0.036	1.8 (1, 5.5)	1.9 (1.2, 5.5)	1.9 (1.1, 5.5)	0.510
TC (mmol/L)	4.1 (2.8, 6.4)	3.2 (2.2, 4.1)	3.9 (2.7, 5.9)	0.001	3.9 (2.6, 5.9)	4 (2.7, 5.8)	3.9 (2.7, 5.9)	0.636
ALT (U/L)	48 (24.5, 108)	49 (26.5, 138.5)	48 (25, 109.8)	0.356	42.5 (24, 93.5)	65.5 (28, 146)	48 (25, 109.8)	0.031
BUN (mmol/L)	7.4 (4.5, 13.5)	10.7 (7.8, 22.3)	7.8 (4.7, 14.2)	< 0.001	8.3 (5.2, 17.8)	7.2 (4.6, 10.5)	7.8 (4.7, 14.2)	0.028
Cr (umol/L)	88 (60.5, 137.5)	178 (105.5, 317)	95 (62, 160)	< 0.001	92 (61, 160)	96 (65.5, 162.5)	95 (62, 160)	0.714
GLU (mmol/L)	11.5 (7.5, 15.6)	9 (6, 18.2)	11.5 (7.3, 15.6)	0.283	12.2 (8.1, 15.7)	8.7 (7.2, 13.7)	11.5 (7.3, 15.6)	0.006
K^+^ (mmol/L)	4.2 (3.7, 4.6)	4.3 (4, 4.8)	4.2 (3.7, 4.6)	0.156	4.2 (3.7, 4.6)	4.2 (3.8, 4.7)	4.2 (3.7, 4.6)	0.879
Ca^2+^ (mmol/L)	1.9 (1.7, 2)	1.9 (1.7, 1.9)	1.9 (1.7, 2)	0.598	1.9 (1.7, 1.9)	1.9 (1.8, 2)	1.9 (1.7, 2)	0.037
Interventions^b^								
VA				< 0.001				0.220
No	193 (72.3)	13 (33.3)	206 (67.3)		150 (69.4)	56 (62.2)	206 (67.3)	
Yes	74 (27.7)	26 (66.7)	100 (32.7)		66 (30.6)	34 (37.8)	100 (32.7)	
Antibiotics				1.000				0.582
No	32 (12)	4 (10.3)	36 (11.8)		24 (11.1)	12 (13.3)	36 (11.8)	
Yes	235 (88)	35 (89.7)	270 (88.2)		192 (88.9)	78 (86.7)	270 (88.2)	
Corticosteroid				0.005				0.145
No	206 (77.2)	22 (56.4)	228 (74.5)		166 (76.9)	62 (68.9)	228 (74.5)	
Yes	61 (22.8)	17 (43.6)	78 (25.5)		50 (23.1)	28 (31.1)	78 (25.5)	
IMV				< 0.001				0.274
No	219 (82)	21 (53.8)	240 (78.4)		173 (80.1)	67 (74.4)	240 (78.4)	
Yes	48 (18)	18 (46.2)	66 (21.6)		43 (19.9)	23 (25.6)	66 (21.6)	
CRRT				< 0.001				0.339
No	224 (83.9)	17 (43.6)	241 (78.8)		167 (77.3)	74 (82.2)	241 (78.8)	
Yes	43 (16.1)	22 (56.4)	65 (21.2)		49 (22.7)	16 (17.8)	65 (21.2)	
Surgical treatment				< 0.001				0.552
No	237 (88.8)	26 (66.7)	263 (85.9)		184 (85.2)	79 (87.8)	263 (85.9)	
Yes	30 (11.2)	13 (33.3)	43 (14.1)		32 (14.8)	11 (12.2)	43 (14.1)	
Peritoneal drainage				0.005				0.059
No	146 (54.7)	12 (30.8)	158 (51.6)		104 (48.1)	54 (60)	158 (51.6)	
Yes	121 (45.3)	27 (69.2)	148 (48.4)		112 (51.9)	36 (40)	148 (48.4)	
Complication				0.003				0.495
No	156 (58.4)	13 (33.3)	169 (55.2)		122 (56.5)	47 (52.2)	169 (55.2)	
Yes	111 (41.6)	26 (66.7)	137 (44.8)		94 (43.5)	43 (47.8)	137 (44.8)	
LOS^b^								
Hospital	22 (14, 34)	19 (6.5, 39)	21.5 (14, 35)	0.268	21.5 (14, 35.2)	21.5 (14, 34.8)	21.5 (14, 35)	0.600
ICU	6 (4, 10)	17 (5, 29)	6 (4, 12.8)	< 0.001	6 (4, 12)	6 (3.2, 14.8)	6 (4, 12.8)	0.863
Outcome^a^								0.842
Survivors					189 (87.5)	78 (86.7)	267 (87.3)	
Nonsurvivors					27 (12.5)	12 (13.3)	39 (12.7)	

BMI, Body Mass Index; APACHE II, Acute physiological and Chronic health Evaluation II score; SOFA, sequential organ failure assessment score; COPD, chronic obstructive pulmonary disease; VA, vasoactive agent; IMV, invasive mechanical ventilation; CRRT, continuous renal replacement therapy; LOS, length of stay; ^a^M(Q1, Q3), ^b^*n* (%).

### 3.2 Model construction and evaluation

#### 3.2.1 LASSO regression screening for predictors

**Figure 1:**
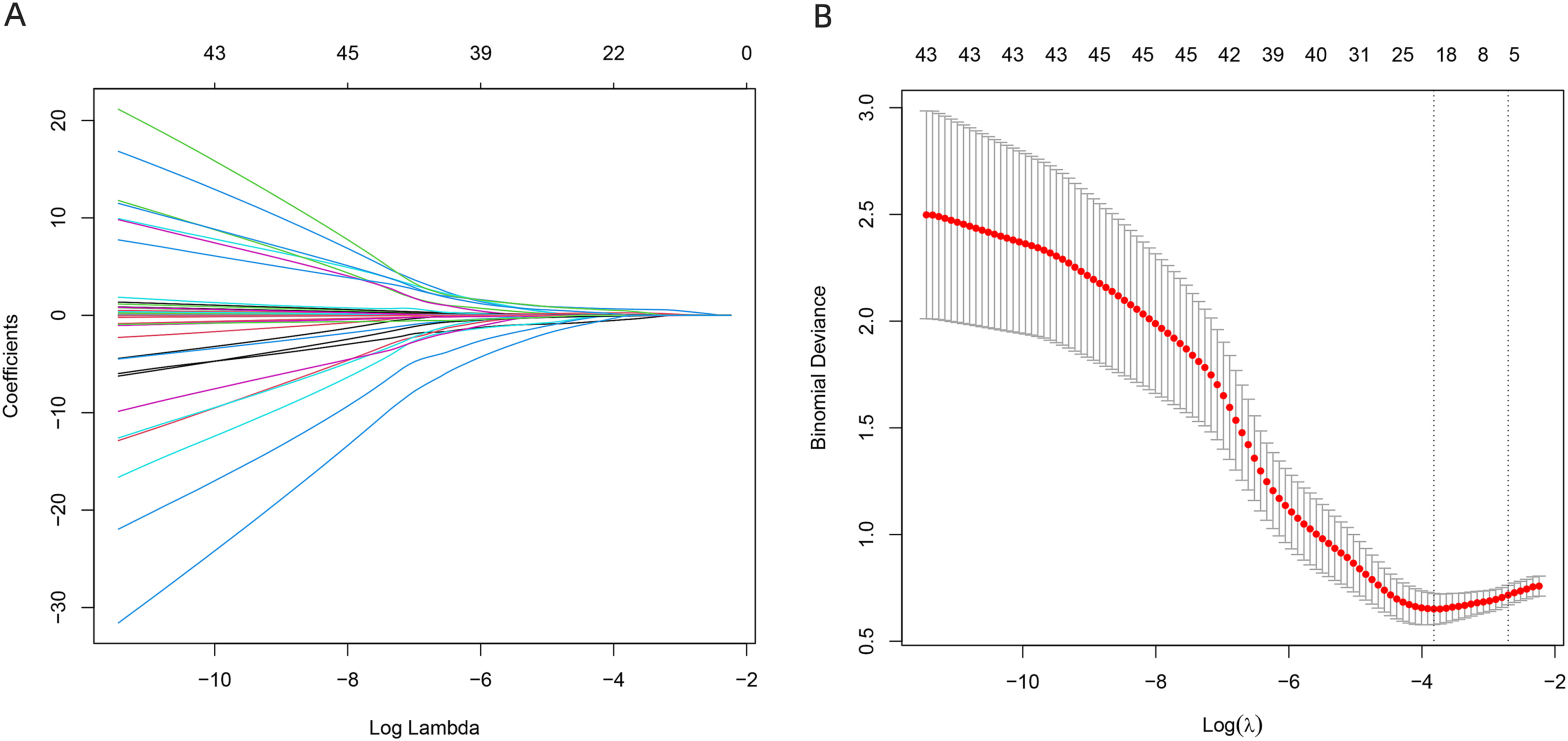
Feature selection using the LASSO regression model. **A** is the LASSO curve; **B** is the process of screening the most suitable *λ* through the 5-fold cross-validation method in the LASSO model.

All variables were included in the LASSO logistic regression model for feature selection. The regularization parameter was determined using the lambda.1SE criterion, which selects the largest lambda within one standard error of the minimum cross-validated error, promoting a more parsimonious model. The selected predictors were: APACHE II score, APTT, Alb, BUN, Cr, use of vasoactive agents, and ICU stay duration (Figures 1A,B).

#### 3.2.2 Multivariate logistic regression analysis

The predictors identified by LASSO regression were incorporated into multivariate logistic regression analysis. The results indicated that these predictors were independent risk factors for mortality in AP patients admitted to the ICU (Table 2).

**Table 2:** Multivariate logistic regression analysis of AP mortality.

Variables	*B*	S. E	Wald	dF	*p*	OR	95% CI	
							Lower	Upper
APACHE II	0.089	0.044	4.079	1	0.043	1.093	1.003	1.192
APTT	0.020	0.014	2.051	1	0.152	1.020	0.993	1.048
Alb	−0.085	0.036	5.637	1	0.018	0.919	0.856	0.985
BUN	0.058	0.029	4.014	1	0.045	1.059	1.001	1.121
Cr	0.000	0.001	0.111	1	0.739	1.000	0.998	1.003
VA	1.304	0.562	5.39	1	0.020	3.682	1.225	11.069
LOS of ICU	0.028	0.015	3.22	1	0.073	1.028	0.997	1.060

APACHE II, Acute Physiology and Chronic Health Evaluation; APTT, Activated Partial Thromboplastin Time; Alb, Albumin; BUN, Blood Urea Nitrogen; Cr, Creatinine; VA, Vasoactive Agents; LOS, Length of Stay; ICU, Intensive Care Unit.

#### 3.2.3 Construction and evaluation of the model

**Figure 2:**
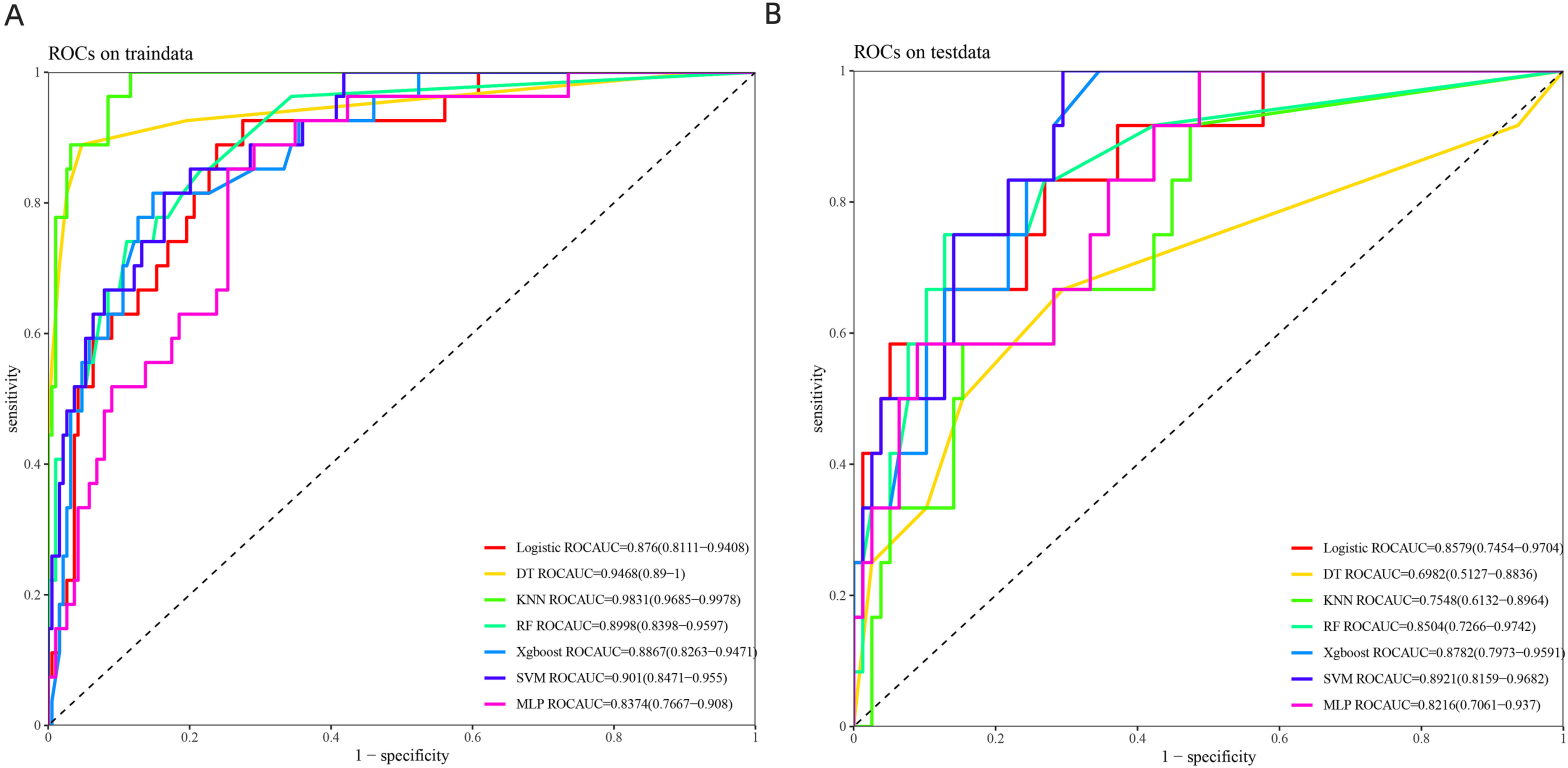
ROC curves of the 7 prediction models in the training set **(A)** and the validation set **(B)**.

The receiver operating characteristic (ROC) curves for the seven prediction models (DT, RF, XGBoost, SVM, MLP, KNN, and LR) were plotted for both the training and validation sets to assess their ability to predict mortality risk in AP patients (Figures 2A,B). In the training set, the area under the curve (AUC) values were as follows: DT (0.947), RF (0.9), XGBoost (0.887), SVM (0.901), MLP (0.837), KNN (0.983), and LR (0.876). In the validation set, the AUC values were: DT (0.698), RF (0.85), XGBoost (0.878), SVM (0.892), MLP (0.822), KNN (0.755), and LR (0.858) (Figures 2A,B; Table 3). Although DT and KNN demonstrated high sensitivity and specificity in the training set, their performance was suboptimal in the validation set, with AUC values of 0.698 and 0.755, respectively. Although SVM showed slightly lower sensitivity, specificity, and AUC values than DT and KNN in the training set (AUC values of 0.901, 0.947, and 0.983), it had the highest AUC in the validation set, with an AUC of 0.892. The AUC values and prediction performance for each model are compared in Table 3. Based on AUC, accuracy, specificity, and sensitivity, SVM emerged as the most robust model.

**Table 3:** Predictive performance of different models.

ML model	Training set						Validation set					
	AUC	Accuracy	Sensitivity	Specificity	F1	Brier	AUC	Accuracy	Sensitivity	Specificity	F1	Brier
DT	0.947	0.944	0.889	0.952	0.8	0.035	0.698	0.8	0.5	0.846	0.4	0.135
RF	0.9	0.792	0.852	0.783	0.505	0.079	0.85	0.744	0.833	0.731	0.465	0.093
XGBoost	0.887	0.847	0.815	0.852	0.571	0.08	0.878	0.756	0.75	0.756	0.45	0.086
SVM	0.901	0.806	0.852	0.799	0.523	0.072	0.892	0.733	0.917	0.705	0.478	0.086
MLP	0.837	0.731	0.889	0.709	0.453	0.143	0.822	0.656	0.75	0.641	0.367	0.147
KNN	0.983	0.898	1	0.884	0.711	0.04	0.755	0.711	0.667	0.718	0.381	0.035
LR	0.876	0.75	0.926	0.725	0.481	0.081	0.858	0.722	0.833	0.705	0.444	0.084

ML: machine learning; DT, Decision Tree; RF, Random Forest; XGBoost, Extreme Gradient Boosting; SVM, Support Vector Machines; MLP, Multi-Layer Perceptron; KNN, K-Nearest Neighbors; LR, Logistic Regression.

**Figure 3:**
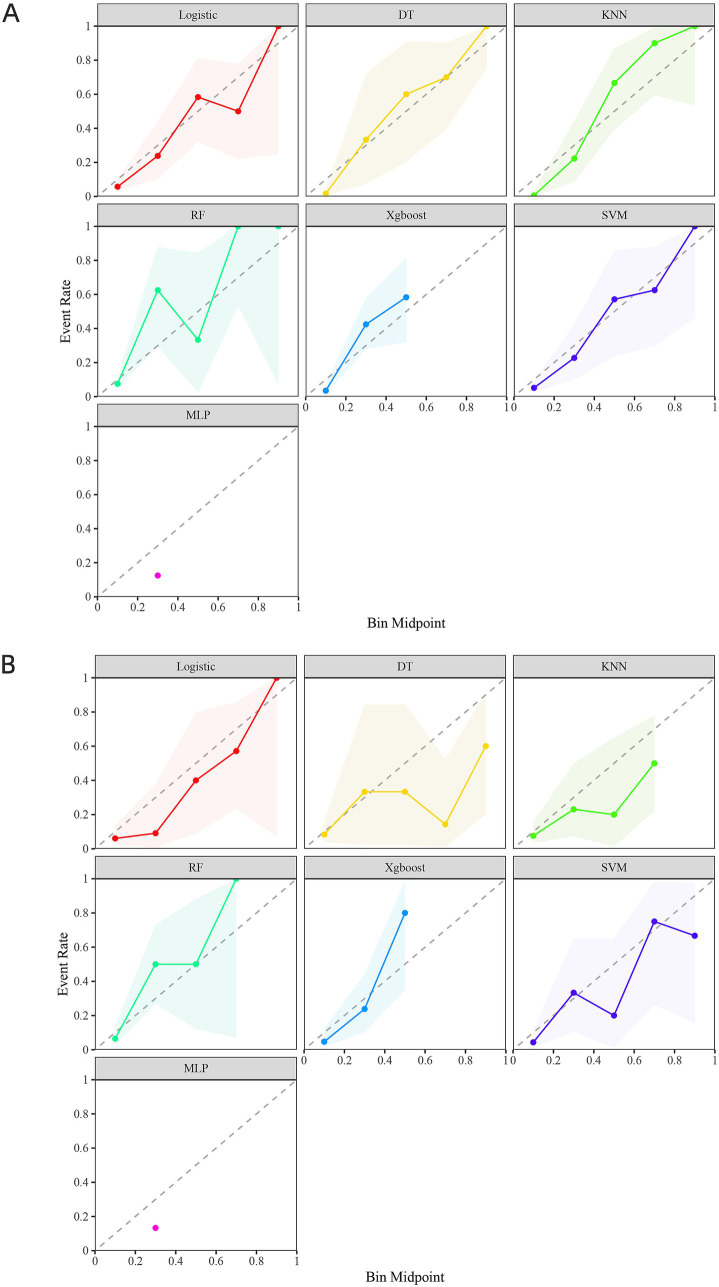
shows the calibration curves of seven prediction models in the training set **(A)** and the validation set **(B)**.

**Figure 4:**
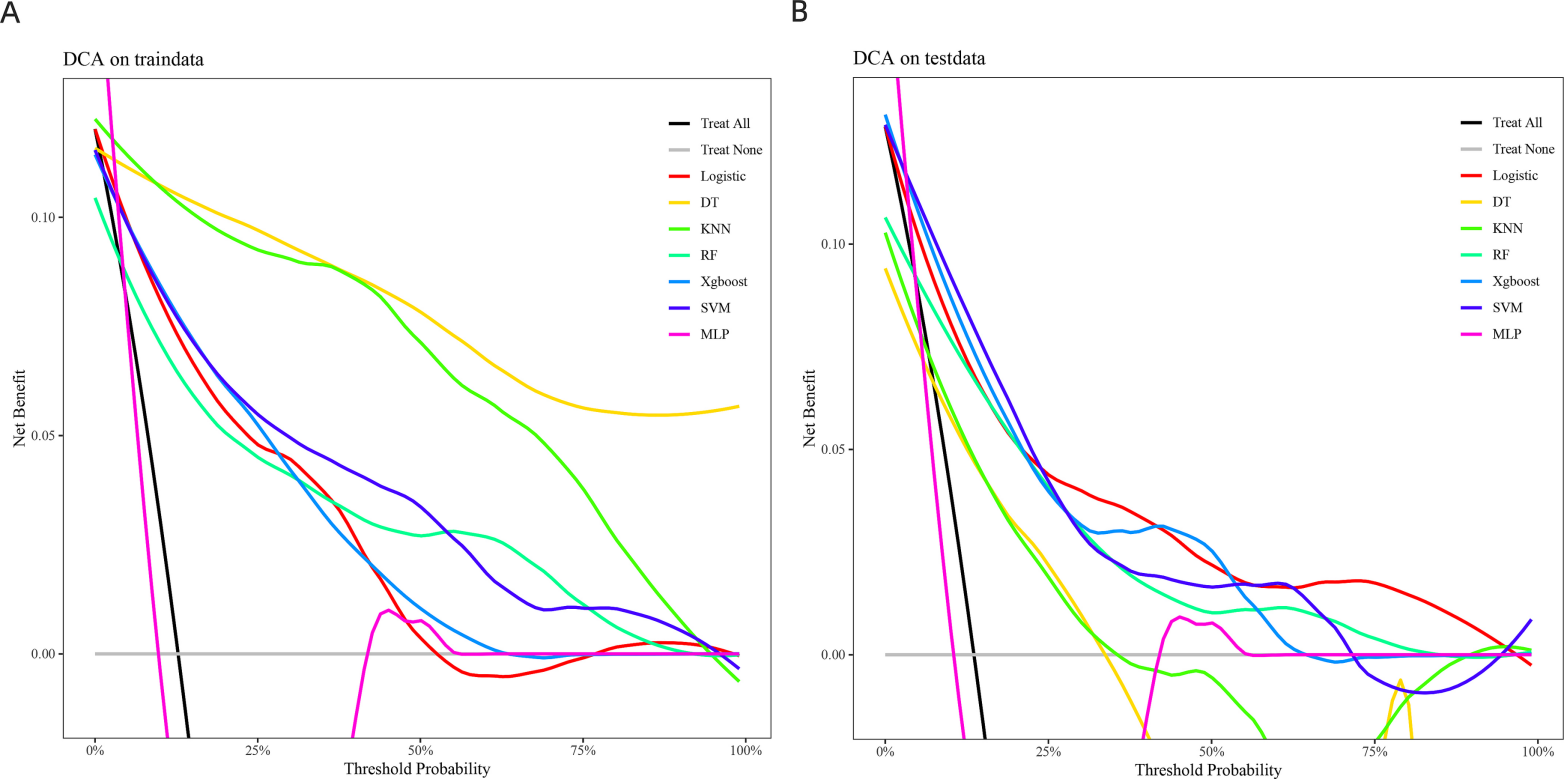
shows the decision curves of the seven prediction models in the training set **(A)** and the validation set **(B)**.

The calibration curves for each prediction model indicated that the DT and SVM models provided stable predictions in the training set, though their performance was slightly less accurate in the validation set. In contrast, the LR model performed better in the validation set (Figures 3A,B). The decision curve analysis (DCA) revealed that the KNN model provided the greatest clinical benefit in the training set, while the SVM model offered more clinical benefit in the validation set (Figures 4A,B).

#### 3.2.4 Visualization by SHAP

**Figure 5:**
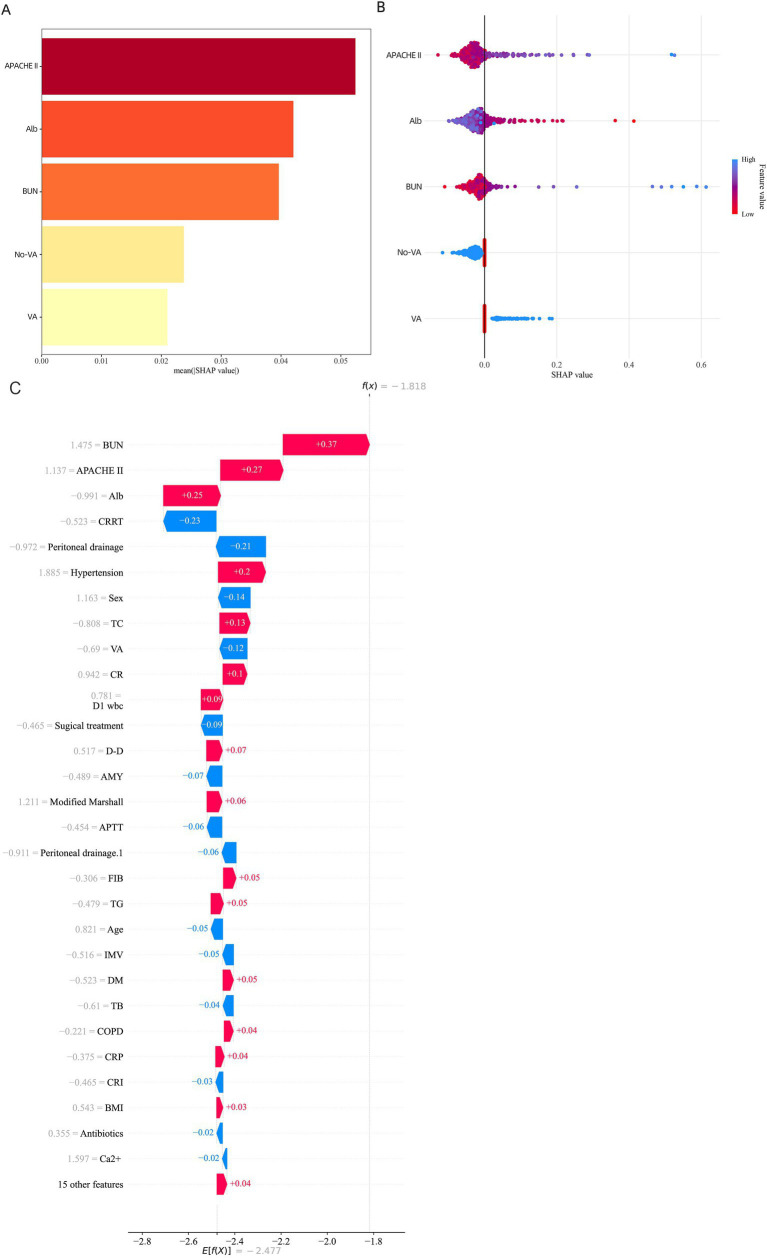
SHAP Feature Importance Summary diagram. **(A)** SHAP waterfall plot for a representative case with a high predicted probability. **(B)** SHAP waterfall plot for a representative case with a low predicted probability. Key features such as APACHE II score, albumin, BUN, and vasoactive agent usage contributed most significantly to individual predictions. **(C)** SHAP waterfall plot illustrating the local interpretability of the model prediction for an individual patient with acute pancreatitis.

In addition to model selection, we employed the SHAP algorithm to explain the prediction model. Figures 5A,B display the feature importance rankings for the SVM model. The four most significant predictors were: APACHE II score, albumin (Alb), urea nitrogen (BUN), and the use of vasoactive agents. Higher APACHE II scores, elevated BUN levels, and lower albumin levels were associated with higher mortality in AP patients, while the use of vasoactive agents also increased mortality risk (Figures 5A,B). To elucidate the interpretability of our machine learning model, we utilized SHAP (SHapley Additive exPlanations) to analyze the contribution of individual variables to the predicted outcome. Figure 5C illustrates a SHAP waterfall plot for a representative patient with acute pancreatitis, demonstrating how each clinical feature shifted the model prediction relative to the baseline.

Among the most influential predictors increasing the model’s output were elevated blood urea nitrogen (BUN) (+0.37), higher APACHE II score (+0.27), and decreased serum albumin (Alb) levels (+0.25). These features collectively contributed to an increased risk of adverse outcome. In contrast, variables such as receiving continuous renal replacement therapy (CRRT) (−0.23) and undergoing peritoneal drainage (−0.21) were associated with a reduction in predicted risk.

The cumulative SHAP values shifted the model’s log-odds output from the base value to a final prediction score of f(x) = −1.81, corresponding to a lower estimated probability of poor prognosis. This individualized explanation highlights the model’s capacity to integrate complex clinical variables and produce interpretable, patient-specific risk predictions.

## 4 Discussion

In this study, LASSO regression was used to identify key variables, resulting in the selection of seven predictors: APACHE II score, APTT, albumin, BUN, creatinine, use of vasoactive agents, and ICU length of stay. Seven machine learning models were developed and validated to predict in-hospital mortality among ICU patients with acute pancreatitis (AP). The SVM model demonstrated superior predictive efficiency compared to the other six models. To further assess the predictive efficiency of the SVM model, a SHAP feature importance graph was generated, illustrating the model’s workings (11). The Shapley value, derived from game theory, quantifies the contribution of each feature to the model’s predictions, highlighting the influence of various features on the model’s output. This approach reveals the non-linear relationships between features and predicted outcomes, thereby ensuring both model performance and clinical interpretability (12).

The SVM algorithm is known for its robustness, capable of solving non-linear problems and improving predictive performance. Unlike statistical models, which only capture linear relationships between features and outcomes, machine learning techniques can model complex, non-linear relationships, enhancing prediction efficiency. However, this improvement in predictive performance often compromises model interpretability, as machine learning models are often considered “black boxes”—we can observe the inputs and outputs, but the processes between them remain opaque (11). To address this, SHAP was employed to explain the model. Shapley values offer a solution from game theory, measuring the contribution of each feature to the model’s predictions, elucidating the role of different features in determining output, and revealing the non-linear relationship between features and outcomes, ensuring the model’s performance and clinical interpretability (12).

In recent years, the SVM algorithm has been applied in some studies on AP. Researchers have employed various machine learning algorithms, including decision trees, random forests, logistic regression, SVM, CatBoost, and XGBoost (13). In our study, seven machine learning models were used to predict the prognosis of ICU patients with AP, and SVM demonstrated the best performance. The SHAP feature importance graph was utilized to explain the model, enhancing the reliability of the results.

This study identified several independent risk factors for mortality in ICU patients with AP: APACHE II score, APTT, albumin, urea nitrogen, serum creatinine, use of vasoactive agents, and ICU length of stay. The SHAP feature importance map revealed that the APACHE II score was the most significant predictor. The APACHE II score is a non-specific scoring system widely used in ICUs to assess disease severity and prognosis. Previous studies have demonstrated that APACHE II is an independent risk factor for predicting pancreatic necrosis, organ failure, and mortality in AP patients (7, 14), which aligns with the findings of this study.

In the case of severe acute pancreatitis (SAP), the activation of inflammatory factors leads to vascular endothelial damage, which triggers the release of tissue factor. This factor activates the coagulation system, initiating the coagulation cascade and disrupting the balance between coagulation and anticoagulation, resulting in coagulopathy and eventually microcirculatory disturbances, both in the pancreas and throughout the body (15). This may explain why APTT, used as an indicator of coagulation function in this study, serves as an independent risk factor for mortality in ICU patients with SAP.

Albumin, a multifunctional protein synthesized by the liver, plays essential roles in maintaining plasma colloid osmotic pressure, immune regulation, inflammation inhibition, and antioxidation (16–18). Hypoalbuminemia in early SAP has been associated with poor prognosis, and timely albumin infusion can reduce mortality in SAP patients with hypoalbuminemia (19). The mechanisms underlying this include reduced albumin synthesis due to inflammatory factor release, albumin loss from capillary leakage caused by endothelial cell damage, and decreased protein intake due to fasting during SAP (18, 20). Our study found that albumin was an independent risk factor for mortality in ICU patients with SAP, with albumin levels being significantly lower in the mortality group compared to the survival group.

SAP often progresses to multi-organ dysfunction, including acute kidney injury (AKI), particularly in the kidneys, which are vulnerable to damage during SAP. AKI occurs in up to 70% of SAP patients (21), with elevated urea nitrogen and creatinine levels serving as important prognostic indicators (22–24). Blood creatinine levels, unaffected by changes in blood volume, are more indicative of organ damage (23). The pathophysiology of AKI in SAP remains unclear but may involve hypovolemia, uncontrolled inflammatory responses, microcirculatory disturbances, and the toxic effects of substances released by necrotic pancreatic tissue (25).

Additionally, this study found that the use of vasoactive agents and ICU length of stay were independent risk factors for mortality in AP patients. Vasoactive agents are commonly used in patients with shock, particularly septic shock, which often complicates AP. The combination of AP and septic shock is a marker of disease progression and increased mortality risk (26). Patients with AP typically experience two peaks in mortality: the first within 2 weeks due to inflammatory response and organ damage, and the second between 2 and 4 weeks, when sepsis and septic shock predominate. This later phase is marked by local complications, such as pancreatic necrosis and infection, and systemic complications, such as multiple organ failure, which can lead to further deterioration and death (27). The use of vasopressors and the prolonged ICU stay, often due to multi-drug-resistant bacterial infections and other complications, significantly contribute to increased mortality and hospital costs.

The limitations of this study include its single-center retrospective design, which may introduce selection bias, and the absence of certain clinical variables, which could impact the findings. Additionally, the LASSO logistic regression model used for feature selection does not account for potential interactions among predictors, and its selection results may be unstable when collinearity exists. Furthermore, LASSO assumes linear relationships between predictors and outcomes, which may oversimplify the complexity of real-world clinical data. Future studies with larger multicenter datasets should consider including interaction terms and exploring alternative or complementary feature selection approaches to enhance model performance and interpretability.

## 5 Conclusion

In summary, this study created a machine learning model that is both interpretable and clinically relevant for predicting in-hospital mortality among ICU patients suffering from acute pancreatitis. Among the seven models tested, SVM demonstrated the best overall performance, balancing accuracy, calibration, and clinical utility. SHAP-based interpretation revealed that higher APACHE II scores, lower albumin levels, prolonged ICU stays, use of vasoactive agents, renal dysfunction markers (BUN, creatinine), and coagulation abnormalities (APTT) were the most influential predictors of mortality. This interpretable model may assist clinicians in early identification of high-risk patients, enabling timely and targeted interventions to improve outcomes in critical care settings.

## Data Availability

The original contributions presented in the study are included in the article/supplementary material, further inquiries can be directed to the corresponding author.
